# Mind the Gap: Bit-vector Interpolation recast over Linear Integer Arithmetic

**DOI:** 10.1007/978-3-030-45190-5_5

**Published:** 2020-03-13

**Authors:** Takamasa Okudono, Andy King

**Affiliations:** 8 Johannes Kepler University, Linz, Austria; 9 University of Birmingham, Birmingham, UK; 10 National Institute of Informatics, Tokyo, Japan; 11 The Graduate University for Advanced Studies (SOKENDAI), Tokyo, Japan; 12 University of Kent, Canterbury, UK

## Abstract

Much of an interpolation engine for bit-vector (BV) arithmetic can be constructed by observing that BV arithmetic can be modeled with linear integer arithmetic (LIA). Two BV formulae can thus be translated into two LIA formulae and then an interpolation engine for LIA used to derive an interpolant, albeit one expressed in LIA. The construction is completed by back-translating the LIA interpolant into a BV formula whose models coincide with those of the LIA interpolant. This paper develops a back-translation algorithm showing, for the first time, how back-translation can be universally applied, whatever the LIA interpolant. This avoids the need for deriving a BV interpolant by bit-blasting the BV formulae, as a backup process when back-translation fails. The new back-translation process relies on a novel geometric technique, called gapping, the correctness and practicality of which are demonstrated.

## 1 Introduction

Given two formulae $A$ and $B$ which are inconsistent, an interpolant for the ordered pair $\langle A, B\rangle$ is a formula $I$ over the variables common to both $A$ and $B$ which is a relaxation of $A$ that is still inconsistent with $B$. For example, when working over the theory of linear inequalities, if $A = (x = y + 1) \wedge (y = 0)$ and $B = (x = z + 2) \wedge (1 \leq z)$ then interpolants for $\langle A, B\rangle$ are $I_1 = (x = 1)$, $I_2 = (x \leq 1)$ and $I_3 = (x < 3)$, ordering by increasing generality. The intuition behind $I_1, I_2$ and $I_3$ is that they are abstractions of $A$ which concisely explain the inconsistency between $A$ and $B$. Interpolation has attracted growing attention over the last decade [26], because of the crucial role it plays in model checking in lazy [18] predicate abstraction [15] and lazy abstraction with interpolants [25], as exemplified in BLAST [5] and IMPACT [25] respectively. In lazy predicate abstraction [25], interpolation is used to synthesise predicates which describe program state. Predicates are added, on demand, to explain why a path through a program cannot reach an error state. In lazy abstraction with interpolants [25], program state is described with unrestricted formulae, rather than merely using predicates, and interpolation is applied to relax sequences of formulae that describe the states down paths which do not error. Interpolation simplify these formulae but increasing the likelihood of covering, again accelerating path exploration. In effect, interpolation is the key abstraction mechanism.

*Context* As solvers for richer theories have evolved so have interpolation engines for these theories, with a notable flurry of activity around one decade ago [10, 11, 19, 20, 23, 24, 30]. However, progress on the important theory of bit-vectors (BV) has been surprisingly slow, the two key works [2, 16] taking opposing approaches. One takes advantage of existing interpolation engines [16] and the another develops a bespoke interpolation engine around lazy reduction [2], which supports bit-vector operations by expanding them, on demand, to Presburger arithmetic [2]. This paper develops the former approach, aiming to use an LIA solver as is.

The central problem in bit-vector interpolation is to construct an interpolant which is compact (one might even say beautiful [1]). Although a pair of inconsistent BV formulae can always be bit-blasted (unfolded) into a pair of inconsistent propositional formulae, it is not always obvious how the resulting propositional interpolant can be folded back into a compact bit-vector (BV) formula to derive a BV interpolant. Interpolation engines over linear integer arithmetic (LIA) have thus been repurposed for BV interpolation [16]. First, operations on bit-vectors are reformulated as LIA formulae. An interpolant over LIA is then reinterpreted as a candidate interpolant for a pair of BV formulae. Because of wrap-around, LIA does not necessarily align with BV arithmetic, hence the LIA interpolant is adopted as a BV interpolant only if it passes a (unsatisfiability) check over bit-vectors. This checks that the interpolant relaxes the first BV formula of the pair and yet is still inconsistent with the second. If the candidate fails the check, then the two BV formulae are bit-blasted to recover a propositional interpolant, albeit one which looses the high-level structure of bit-vectors, and therefore is not compact. This approach is promising: it exploits robust off-the-shelf LIA interpolation [17] yet is compromised by the quality of the interpolants which follow from bit-blasting.

*Contribution* This paper plugs this gap, addressing the issue of interpolant quality by developing a new, principled encoding LIA formulae into BV formulae which does not enlarge the bit-width of the BV formulae. This ensures that the interpolant is still drawn from the language used to define BV formulae. We show that a naïve encoding of an LIA inequality as a BV inequality can give a formula which has a completely different meaning from LIA inequality: the BV inequality can have solutions not admitted by the LIA inequality and vice versa. Moreover, we illustrate how a straightforward encoding of a single LIA inequality can require many BV inequalities, which compromises the quality of a BV interpolant. We therefore propose a technique, which we call gapping, which adds range constraints to LIA inequality which reduces the LIA inequality into two or three LIA systems the solutions of which are amenable to compact BV representation. The term gapping reflects a geometric interpretation of this transformation which introduces a gap[4] between the solutions of the two LIA systems. We demonstrate the value of this approach with a BV interpolation engine which side-steps bit-blasting (and the complexity of providing bit-level

[4] The title of the paper alludes to both this geometric technique, the conceptual gap in previous work, and collaboration which entailed traveling through London.

circuits for arithmetic) and show that the approach usually gives a modest slowdown relative to LIA. We also prove the validity of the BV encoding, and the correctness of the reductions the encoding relies on, though the proofs themselves are omitted here for brevity. To summarise, the contributions of this paper are as follows:

- We show how interpolating theorem provers for LIA can be used to interpolate BV formulae, without recourse to bit-blasting.
- We develop a rigorous theory which explains gapping and proves that the resulting BV interpolant has exactly the same set of models as the LIA interpolant of the two BV formulae.
- We provide evaluation, within the established [6] framework of lazy abstraction with interpolants [25] which demonstrates the practicality of the approach for BV interpolation.

*Use case* Since BV formulae are converted to LIA one might wonder why one cannot work with LIA throughout and avoid BV interpolation all together. First, such an approach would not fit with a layered approach to interpolation [17] where one uses one lightweight theory (eg. uninterpreted functors) and then, if necessary, a more complicated one (eg. LIA) to construct a BV interpolant. BV formulae provide a uniform way expressing interpolants, no matter how they are derived. Second, computing LIA interpolants is complex and it is not surprising that these engines contain subtle[5] bugs. Translating a LIA interpolant back into a BV formula enables interpolants to be validated using a BV solver [3, 7, 27], using the reference (BV) semantics of a program. Moreover, validation need make no assumption on the correctness of a translation between theories. Validation can be performed on-the-fly, as the unwinding tree [25] is constructed, or by translating the complete, stable unwinding tree into its BV counterpart. The BV version can then be validated as a form of post-processing, akin to post-fixpoint validation in abstract interpretation [4, 14].

*Road map* This paper is structured as follows: Section 2 gives the intuition behind boxing and gapping whereas Section 3 argues for the correctness of the approach. Section 4 presents the experimental work. Section 5 presents the related work and section 6 concludes.

## 2 Boxing and Gapping in Pictures

Given a linear inequality $\ell$, we seek to find a bit-vector formula $f$ such that $[\![f]\!]_{\mathsf{BV}} = [\![\ell]\!]_{\mathsf{LIA}}$ where $[\![f]\!]_{\mathsf{BV}}$ and $[\![\ell]\!]_{\mathsf{LIA}}$ are respectively the sets of solutions (models) of $f$ and $\ell$ in the linear integer arithmetic (LIA) and bit-vector (BV) semantics. Ideally $f$ should be compact where we measure size by the number of binary logical connectives in $f$. This section gives the intuition behind two

[5] We refrain from mentioning specific solvers because we do not want to embarrass any particular research team to whom we are grateful.

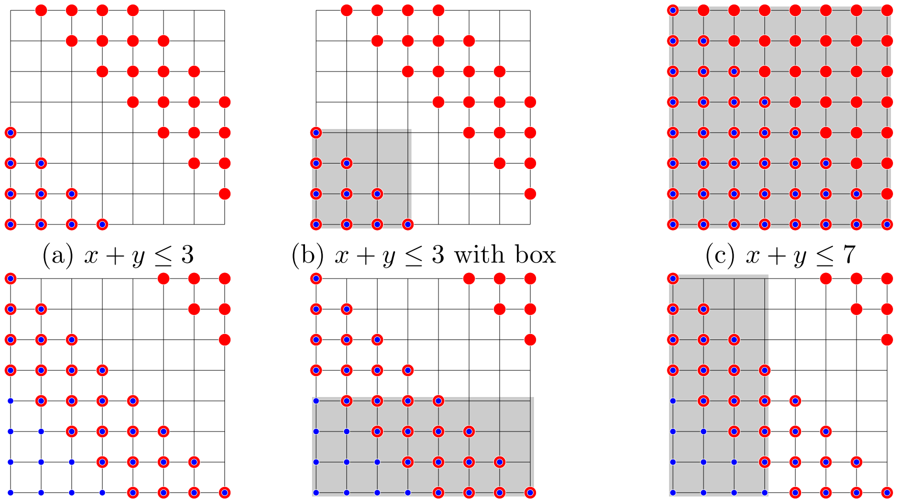

(a) $x + y \leq 3$ (b) $x + y \leq 3$ with box (c) $x + y \leq 7$

(d) $x + y - 4 \leq 3$ (e) $x + y - 4 \leq 3$ with box 1 (f) $x + y - 4 \leq 3$ with box 2

**Fig. 1.** Gapping and boxing for $x + y \leq 3$ and $x + y \leq 7$

techniques, boxing and gapping, and demonstrate how they are used together to construct such an $f$; the sequel provides a more formal development.

To illustrate boxing and gapping, first consider the set of solutions to the inequality $x + y \leq 3$, when interpreted with both the LIA semantics and BV semantics. Figure 1(a) gives the LIA solutions in blue and the BV solutions in red over the non-negative integer grid $\{(x, y) \mid 0 \leq x < 8 \wedge 0 \leq y < 8\}$ using a modulo of 8 for bit-vectors. The solution sets differ on, for instance, $(5, 6)$ since $(5 + 6) \pmod 8 = 3 \leq 3$ but $5 + 6 = 11 \not\leq 3$. It does not generally follow that $[\![f]\!]_{\mathsf{LIA}} \subseteq [\![f]\!]_{\mathsf{BV}}$ as Figure 1(d) illustrates for $f = x + y - 4 \leq 3$. Then $(1, 2) \in [\![x + y - 4 \leq 3]\!]_{\mathsf{LIA}}$ since $1+2-4 = -1 \leq 3$ but $(1, 2) \notin [\![x + y - 4 \leq 3]\!]_{\mathsf{BV}}$ since $(1 + 2 - 4) \pmod 8 = 7 \not\leq 3$.

*Enumeration* A naive approach to finding a formula $f$ such that $[\![f]\!]_{\mathsf{BV}} = [\![\ell]\!]_{\mathsf{LIA}}$ is to enumerate all solutions of $[\![\ell]\!]_{\mathsf{LIA}}$ to then summarise them in a single BV formula. Figure 1(a) illustrates the $4 + 3 + 2 + 1 = 10$ LIA solutions for $\ell = (x + y \leq 3)$ which are summarised in the following BV formula:

$$f_1 = (x = 0 \wedge y = 0) \vee \ldots \vee (x = 0 \wedge y = 3) \vee \ldots \vee (x = 3 \wedge y = 0)$$

This formula has 9 binary disjuncts and 10 binary conjuncts, hence 19 logical connectives in total. A more compact formulation is to cover the blue triangular region of Figure 1(a) with columns as realised with the following BV formula:

$$f_2 = (x = 0 \wedge y \leq 3) \vee \ldots \vee (x = 3 \wedge y \leq 0)$$

Only non-negative solutions on the grid are considered so there is no need to additionally assert $0 \leq y$. This formula has 3 binary disjuncts and 4 binary conjuncts giving and 7 connectives in total.

*Boxing* Observe from Figure 1(a) that the extra solutions of $[\![x + y \leq 3]\!]_{\mathsf{BV}}$ over $[\![x + y \leq 3]\!]_{\mathsf{LIA}}$ stem from overflow. Overflow can be avoided by constraining BV solutions with $x \leq 3$ and $y \leq 3$ which amounts to placing a box (in general a hyper-rectangle) around the LIA solutions, as illustrated in Figure 1(b). This tactic, henceforth called boxing, leads to the following formula:

$$f_3 = (x + y \leq 3 \wedge x \leq 3 \wedge y \leq 3)$$

which requires 2 binary conjuncts.

*Gapping* Figure 1(c) illustrates that in general boxing cannot be applied in isolation because a box around the LIA solutions would not eliminate any extraneous BV solutions. Boxing is successful for Figure 1(b) because of the absence of solutions (a gap) between the LIA solutions inside the box and the BV solutions outside the box. No such gap exists for the box of Figure 1(c). Yet boxing can still be applied by decomposing the inequality $x + y \leq 7$ into two inequalities both of which are amenable to boxing. The construction is based on $[\![x + y \leq 7]\!]_{\mathsf{LIA}} = [\![x + y \leq 3]\!]_{\mathsf{LIA}} \cup [\![4 \leq x + y \wedge x + y \leq 7]\!]_{\mathsf{LIA}} = [\![x + y \leq 3]\!]_{\mathsf{LIA}} \cup [\![0 \leq x + y - 4 \wedge x + y - 4 \leq 3]\!]_{\mathsf{LIA}}$. Recall that boxing alone allows the LIA solutions of $x + y \leq 3$ to be expressed as a BV formula of 2 binary connectives. Thus consider the compound formula $\ell' = (0 \leq x + y - 4 \wedge x + y - 4 \leq 3)$ whose LIA solutions are illustrated in Figure 1(d). Observe that the BV solutions of $\ell'$ can be covered with two rectangles without including the extraneous 6 BV solutions in top right. Then $[\![\ell']\!]_{\mathsf{LIA}} = [\![x + y - 4 \leq 3 \wedge (x \leq 3 \vee y \leq 3)]\!]_{\mathsf{BV}}$ which leads to the complete formula

$$f_3 = (x + y \leq 3 \wedge x \leq 3 \wedge y \leq 3) \vee (x + y - 4 \leq 3 \wedge (x \leq 3 \vee y \leq 3))$$

such that $[\![f_3]\!]_{\mathsf{BV}} = [\![x + y \leq 7]\!]_{\mathsf{LIA}}$. This tactic of artificially introducing a gap, henceforth called gapping, is equally applicable for larger grids too. For instance, working over a modulo of 32 $[\![x + y \leq 31]\!]_{\mathsf{LIA}} = [\![f_4]\!]_{\mathsf{BV}}$ where

$$f_4 = (x + y \leq 15 \wedge x \leq 15 \wedge y \leq 15) \vee (x + y - 16 \leq 15 \wedge (x \leq 15 \vee y \leq 15))$$

## 3 Formal correctness of boxing and gapping

In what follows we consider LIA and BV formulae over an ordered set of variables $\{x_1, \ldots, x_d\}$ for some $d > 1$. We consider bit-vectors of fixed width $w > 1$ and interpret LIA and BV formulae over the product space $\mathbb{M}^d$ where $\mathbb{M} = \{0, 1, 2, \ldots, m - 1\}$ and $m = 2^w$ as follows:

**Definition 1.** Let $\boldsymbol{c}, \boldsymbol{c'} \in \mathbb{Z}^d$ and $b, b' \in \mathbb{Z}$. If $\ell \equiv (\sum_{i=1}^{d} c_i x_i) + b \leq (\sum_{i=1}^{d} c'_i x_i) + b'$ then

$$[\![\ell]\!]_{\mathsf{LIA}} = \left\{ \boldsymbol{x} \in \mathbb{M}^d \middle| \sum_{i=1}^{d} c_i x_i + b \leq \sum_{i=1}^{d} c'_i x_i + b' \right\}$$

$$[\![\ell]\!]_{\mathsf{BV}} = \left\{ \boldsymbol{x} \in \mathbb{M}^d \middle| (\sum_{i=1}^{d} c_i x_i + b) \bmod m \leq (\sum_{i=1}^{d} c'_i x_i + b') \bmod m \right\}$$

Furthermore, the LIA semantics can be lifted from inequalities to LIA formulae by: $[\![f_1 \vee f_2]\!]_{\mathsf{LIA}} = [\![f_1]\!]_{\mathsf{LIA}} \cup [\![f_2]\!]_{\mathsf{LIA}}$, $[\![f_1 \wedge f_2]\!]_{\mathsf{LIA}} = [\![f_1]\!]_{\mathsf{LIA}} \cap [\![f_2]\!]_{\mathsf{LIA}}$ and $[\![\neg f]\!]_{\mathsf{LIA}} = \mathbb{M}^d \setminus [\![f]\!]_{\mathsf{LIA}}$. Likewise for BV formulae.

In the sequel, $\mathbb{N}$ denotes the set of (strictly) positive integers, $\mathbb{R}$ the set of real numbers, and $\mathbb{R}_{\geq 0}$ the set of non-negative real numbers. We extend the floor and ceiling function for the sequences in $\mathbb{R}^d$ in a component-wise manner: $\lfloor \boldsymbol{x} \rfloor_i = \lfloor x_i \rfloor$ and $\lceil \boldsymbol{x} \rceil_i = \lceil x_i \rceil$. If $\boldsymbol{x} \in \mathbb{R}^d$ then $|\boldsymbol{x}| = d$. The partial order $\leq$ on $\mathbb{R}^d$ is defined by $\boldsymbol{x} \leq \boldsymbol{y}$ if and only if $x_i \leq y_i$ for all $i = 1, \ldots, d$.

### 3.1 Boxing

Boxing is founded on the following result and its corollary in which sets of solutions to inequalities which describe hyper-rectangles are pinched, above and below, by inclusions to systems of inequalities with positive, unary coefficients:

**Lemma 1.** Let $d > 1$ and $L \in \mathbb{N}$. Then:

$$\begin{aligned} &\left\{ \boldsymbol{x} \in \mathbb{R}^d_{\geq 0} \middle| \textstyle\sum_{i=1}^d x_i \leq L \cdot (m/2) - 1 \right\} \\ \subseteq &\textstyle\bigcup_{\boldsymbol{p} \in I_d((d-1)(L+1))} \bigcap_{i=1}^d \left\{ \boldsymbol{x} \in \mathbb{R}^d_{\geq 0} \mid x_i < \frac{p_i \cdot (m/2)}{d-1} \right\} \\ \subseteq &\left\{ \boldsymbol{x} \in \mathbb{R}^d_{\geq 0} \middle| \textstyle\sum_{i=1}^d x_i < (L+1) \cdot (m/2) \right\} \end{aligned}$$

where $I_d(n) = \{(i_1, \ldots, i_d) \in \mathbb{N}^d \mid i_1 + \cdots + i_d = n\}$.

**Corollary 1.** Let $d > 1$, $L \in \mathbb{N}$ and $\boldsymbol{c} \in \mathbb{N}^d$. Then:

$$\begin{aligned} &\left\{ \boldsymbol{x} \in \mathbb{Z}^d_{\geq 0} \middle| \textstyle\sum_{i=1}^d c_i x_i \leq L \cdot (m/2) - 1 \right\} \\ \subseteq &\textstyle\bigcup_{\boldsymbol{p} \in I_d((d-1)(L+1))} \bigcap_{j=1}^d \left\{ \boldsymbol{x} \in \mathbb{Z}^d_{\geq 0} \mid x_j \leq \lceil \frac{p_j \cdot (m/2)}{c_i(d-1)} \rceil - 1 \right\} \\ \subseteq &\left\{ \boldsymbol{x} \in \mathbb{Z}^d_{\geq 0} \mid \textstyle\sum_{i=1}^d c_i x_i \leq (L+1) \cdot (m/2) - 1 \right\} \end{aligned}$$

The corollary leads to two types of box constraint: one for LIA and the other, reducing boxing, for BV. Boxing formulae are purely conceptual and are used to reason about correctness; reduced boxing formulae are deployed within BV interpolants.

**Definition 2.** Let $\boldsymbol{c} \in \mathbb{N}^d$, $b \in \mathbb{N}$ and $L \in \mathbb{N}$ be the unique natural number such that $(L-1) \cdot (m/2) \leq b \leq L \cdot (m/2) - 1$. The *boxing* and *reduced boxing* of $\sum_{i=1}^d c_i x_i \leq b$ are formulae defined as follows:

$$\mathrm{box}_{\mathsf{LIA}}(\boldsymbol{c}; b) \equiv \bigvee_{\boldsymbol{p} \in I_d((d-1)(L+1))} \bigwedge_{j=1}^d \left( x_j \leq \lceil \frac{p_j \cdot (m/2)}{c_j(d-1)} \rceil - 1 \right) \quad (1)$$

$$\mathrm{box}_{\mathsf{BV}}(\boldsymbol{c}; b) \equiv \bigvee_{\boldsymbol{p} \in I_d((d-1)(L+1))} \bigwedge_{j=1}^d \left( x_j \leq \min\left( \lceil \frac{p_j \cdot (m/2)}{c_j(d-1)} \rceil - 1, m-1 \right) \right) \quad (2)$$

Given $m$ and $b \in \mathbb{N}$, it is always possible to find a unique $L \in \mathbb{N}$ which satisfies Definition 2 by putting $L = \lfloor \frac{2b}{m} \rfloor + 1$. Then $L - 1 = \lfloor \frac{2b}{m} \rfloor \leq \frac{2b}{m} < \lfloor \frac{2b}{m} \rfloor + 1 = L$ hence $(L-1)(m/2) \leq b < L(m/2)$ whence $(L-1)(m/2) \leq b \leq L(m/2) - 1$ because $b$ and $L(m/2)$ are integral.

One might expect that the cardinality of $I_d((d-1)(L+1))$ becomes large as $d$ or $L$ grow large. Yet $d$ is the number of variables occurring in the LIA interpolant, which is typically small. Furthermore, when $L$ is large, the values of $p$ are also large, so that many terms become equivalent because of the min operation in equation (2) of Definition 2. Thus the number of terms required to define $\mathrm{box}_{\mathsf{BV}}(\boldsymbol{c}; b)$ does not grow excessively large in practice.

The following proposition asserts that the boxing and reduced boxing formulae share the same solution set when interpreted with, respectively, the LIA and BV semantics.

**Proposition 1.** $[\![\mathrm{box}_{\mathsf{LIA}}(\boldsymbol{c}; b)]\!]_{\mathsf{LIA}} = [\![\mathrm{box}_{\mathsf{BV}}(\boldsymbol{c}; b)]\!]_{\mathsf{BV}}$

*Example 1.* To demonstrate this equivalence, consider again $x + y \leq 3$ for $m = 8$. Then put $L = \lfloor 6/8 \rfloor + 1 = 1$ and $I_2((d-1)(L+1)) = I_2(2) = \{\langle 1, 1\rangle\}$. Observe $\mathrm{box}_{\mathsf{LIA}}(\langle 1, 1\rangle; 3) = \mathrm{box}_{\mathsf{BV}}(\langle 1, 1\rangle; 3)$ since

$$\mathrm{box}_{\mathsf{LIA}}(\langle 1, 1\rangle; 3) = (x \leq \lceil 4/1 \rceil - 1 = 3) \wedge (y \leq \lceil 4/1 \rceil - 1 = 3)$$

$$\mathrm{box}_{\mathsf{BV}}(\langle 1, 1\rangle; 3) = (x \leq \min(3, 7) = 3) \wedge (y \leq \min(3, 7) = 3)$$

*Example 2.* Although $[\![\mathrm{box}_{\mathsf{LIA}}(\boldsymbol{c}; b)]\!]_{\mathsf{LIA}} = [\![\mathrm{box}_{\mathsf{BV}}(\boldsymbol{c}; b)]\!]_{\mathsf{BV}}$, it does not necessarily follow that $[\![\mathrm{box}_{\mathsf{LIA}}(\boldsymbol{c}; b)]\!]_{\mathsf{LIA}} = [\![\mathrm{box}_{\mathsf{LIA}}(\boldsymbol{c}; b)]\!]_{\mathsf{BV}}$. To illustrate, consider $x + y \leq 7$ for $d = 2$ and $m = 4$. Thus $\boldsymbol{c} = \langle 1, 1\rangle$ and $b = 7$. Then $L = \lfloor 14/4 \rfloor + 1 = 4$ and $I_2((d-1)(L+1)) = I_2(5) = \{\langle 1, 4\rangle, \langle 2, 3\rangle, \langle 3, 2\rangle, \langle 4, 1\rangle\}$ hence

$$\begin{aligned}\mathrm{box}_{\mathsf{LIA}}(\boldsymbol{c}; b) = {} & (x \leq 1 \wedge y \leq 7) \vee (x \leq 3 \wedge y \leq 5) \vee \\ & (x \leq 5 \wedge y \leq 3) \vee (x \leq 7 \wedge y \leq 1)\end{aligned}$$

Therefore $[\![\mathrm{box}_{\mathsf{LIA}}(\boldsymbol{c}; b)]\!]_{\mathsf{LIA}} = \mathbb{M}^2$ but $(2, 2) \notin [\![\mathrm{box}_{\mathsf{LIA}}(\boldsymbol{c}; b)]\!]_{\mathsf{BV}}$.

The following lemma shows that the solution sets for boxing grow monotonically as the constant of the inequality is relaxed.

**Lemma 2.** If $b \leq b'$ then $[\![\mathrm{box}_{\mathsf{LIA}}(\boldsymbol{c}; b)]\!]_{\mathsf{LIA}} \subseteq [\![\mathrm{box}_{\mathsf{LIA}}(\boldsymbol{c}; b')]\!]_{\mathsf{LIA}}$.

The following results explains how to augment an inequality with a box so as to align its BV semantics with its LIA semantics.

**Theorem 1 (boxing without gapping).** *Let $\boldsymbol{c} \in \mathbb{N}^d$ and $b \in \mathbb{N}$. If $b < m/2$ then*

$$[\![\sum_{i=1}^{d} c_i x_i \leq b]\!]_{\mathsf{LIA}} = [\![(\sum_{i=1}^{d} c_i x_i \leq b) \wedge \mathrm{box}_{\mathsf{BV}}(\boldsymbol{c}; b)]\!]_{\mathsf{BV}}$$

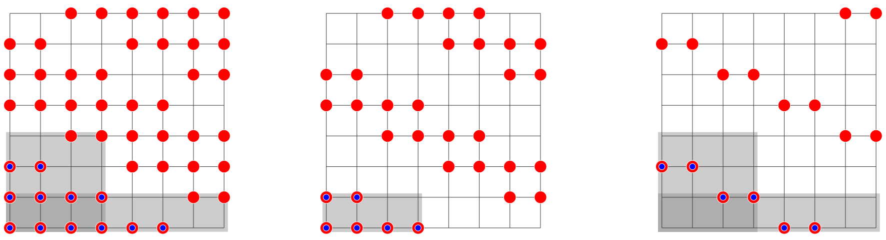

(a) $x + 2y \leq 5$ with boxes (b) $x + 2y \leq 3$ with box (c) $0 \leq x + 2y - 4 \leq 1$ with boxes

**Fig. 2.** Gapping and boxing for $x + 2y \leq 5$

Observe that the result requires $b < m/2$. In this circumstance $L = \lfloor 2b/m \rfloor + 1 = 1$ and number of logical connectives in $\text{box}_{\text{BV}}(\boldsymbol{c}; b)$ is determined by the cardinality of the set $I_d((d-1)(L+1)) = I_d(2(d-1))$, which is given below:

| $d$ | $2(d-1)$ | $I_d(2(d-1))$ | $\lvert I_d(2(d-1)) \rvert$ |
|---|---|---|---|
| 2 | 2 | $\Pi(\langle 1, 1 \rangle)$ | 1 |
| 3 | 4 | $\Pi(\langle 1, 1, 2 \rangle)$ | 3 |
| 4 | 6 | $\Pi(\langle 1, 1, 1, 3 \rangle) \cup \Pi(\langle 1, 1, 2, 2 \rangle)$ | 10 |
| 5 | 8 | $\Pi(\langle 1, 1, 1, 1, 4 \rangle) \cup \Pi(\langle 1, 1, 1, 2, 3 \rangle) \cup \Pi(\langle 1, 1, 2, 2, 2 \rangle)$ | 35 |

where $\Pi(v)$ denote the set of permutations of the vector $v$. For $d = 4$, $\text{box}_{\text{BV}}(\boldsymbol{c}; b)$ thus requires $10(d-1) = 30$ binary conjunctions and $10 - 1 = 9$ disjunctions.

### 3.2 Boxing and Gapping

*Example 3.* Consider $[\![x + 2y \leq 5]\!]_{\text{BV}}$ and $[\![x + 2y \leq 5]\!]_{\text{LIA}}$ for $m = 8$ as shown in Figure 2(a). Observe

$$\text{box}_{\text{BV}}(\langle 1, 2 \rangle; 5) = (x \leq 3 \wedge y \leq 3) \vee (x \leq 7 \wedge y \leq 1)$$

which is illustrated by the two grey rectangles. Hence $\langle 2, 3 \rangle \notin [\![x + 2y \leq 5]\!]_{\text{LIA}}$ but $\langle 2, 3 \rangle \in [\![x + 2y \leq 5 \wedge \text{box}_{\text{BV}}(\langle 1, 2 \rangle; 5)]\!]_{\text{BV}}$ therefore using boxing alone is not sufficient to encode the LIA inequality $x + 2y \leq 5$.

*Example 4.* Yet the LIA inequality $x + 2y \leq 5$ can be decomposed as follows:

$$\begin{aligned} [\![x + 2y \leq 5]\!]_{\text{LIA}} &= [\![x + 2y \leq 3]\!]_{\text{LIA}} \cup [\![4 \leq x + 2y \leq 5]\!]_{\text{LIA}} \\ &= [\![x + 2y \leq 3]\!]_{\text{LIA}} \cup [\![0 \leq x + 2y - 4 \leq 1]\!]_{\text{LIA}} \end{aligned}$$

Figures 2(b, c) illustrates boxing for $x + 2y \leq 3$ and $0 \leq x + 2y - 4 \leq 1$ where:

$$\begin{aligned} [\![x + 2y \leq 3]\!]_{\text{LIA}} &= [\![x + 2y \leq 3 \wedge \text{box}_{\text{BV}}(\langle 1, 2 \rangle; 3)]\!]_{\text{BV}} \\ &= [\![x + 2y \leq 3 \wedge (x \leq 3 \wedge y \leq 1)]\!]_{\text{BV}} \end{aligned}$$

Observe from Figure 2(c) that

$$[\![0 \leq x + 2y - 4 \leq 1]\!]_{\mathsf{LIA}} = [\![0 \leq x + 2y - 4 \leq 1]\!]_{\mathsf{BV}} \cap [\![\mathrm{box}_{\mathsf{BV}}(\langle 1, 2\rangle; 5)]\!]_{\mathsf{BV}}$$

and moreover $0 \bmod 8 = 0 \leq (x + 2y - 4) \bmod 8$ for all $(x, y) \in \mathbb{M}^2$ thus

$$[\![0 \leq x + 2y - 4 \leq 1]\!]_{\mathsf{LIA}} = [\![x + 2y - 4 \leq 1 \wedge \mathrm{box}_{\mathsf{BV}}(\langle 1, 2\rangle; 5)]\!]_{\mathsf{BV}}$$

therefore cumulatively $[\![x + 2y \leq 5]\!]_{\mathsf{LIA}} = [\![\varphi_1 \vee \varphi_2]\!]_{\mathsf{BV}}$ where

$$\begin{aligned} \varphi_1 &= [x + 2y \leq 3 \qquad \wedge (x \leq 3 \wedge y \leq 1)] \\ \varphi_2 &= [x + 2y - 4 \leq 1 \wedge ((x \leq 3 \wedge y \leq 3) \vee (x \leq 7 \wedge y \leq 1))] \end{aligned}$$

The general rule of the separation of the given inequality and the boxing is shown in this theorem:

**Theorem 2 (boxing with gapping).** *Let* $\boldsymbol{c} \in \mathbb{N}^d$ *and* $b \in \mathbb{N}$. $[\![\sum_{i=1}^{d} c_i x_i \leq b]\!]_{\mathsf{LIA}} = [\![\phi_0 \vee \phi_1 \vee \phi_2]\!]_{\mathsf{BV}}$ *where* $S = \lfloor b/(m/2) \rfloor$ *and*

$$\begin{aligned} \phi_0 &\equiv \left(\textstyle\sum_{i=1}^{d} c_i x_i - (S-2)(m/2) \leq m/2 - 1\right) \wedge \mathrm{box}_{\mathsf{BV}}(\boldsymbol{c}; (S-1)(m/2) - 1) \\ \phi_1 &\equiv \left(\textstyle\sum_{i=1}^{d} c_i x_i - (S-1)(m/2) \leq m/2 - 1\right) \wedge \mathrm{box}_{\mathsf{BV}}(\boldsymbol{c}; S(m/2) - 1) \\ \phi_2 &\equiv \left(\textstyle\sum_{i=1}^{d} c_i x_i - S(m/2) \leq b \bmod (m/2)\right) \wedge \mathrm{box}_{\mathsf{BV}}(\boldsymbol{c}; b) \end{aligned}$$

**Corollary 2 (boxing and gapping with simplification).** *If* $\lfloor b/(m/2) \rfloor = 1$ *or* $b \bmod m = m/2 - 1$ *then* $[\![\sum_{i=1}^{d} c_i x_i \leq b]\!]_{\mathsf{LIA}} = [\![\phi_1 \vee \phi_2]\!]_{\mathsf{BV}}$.

*Example 5.* Let $m = 8$ and consider again $x + 2y \leq 5$ so that $\boldsymbol{c} = \langle 1, 2\rangle$. Then $S = \lfloor 5/4 \rfloor = 1$ and, applying corollary 2, $[\![x + 2y \leq 5]\!]_{\mathsf{LIA}} = [\![\phi_1 \vee \phi_2]\!]_{\mathsf{BV}}$ where

$$\begin{aligned} \phi_1 &\equiv (x + 2y - 0 \cdot 4 \leq 4 - 1) \qquad \wedge \mathrm{box}_{\mathsf{BV}}(\boldsymbol{c}; 1 \cdot 4 - 1) = \varphi_1 \\ \phi_2 &\equiv (x + 2y - 1 \cdot 4 \leq 5 \bmod 4) \wedge \mathrm{box}_{\mathsf{BV}}(\boldsymbol{c}; 5) \qquad\quad = \varphi_2 \end{aligned}$$

aligning with the intuition given in example 4.

*Example 6.* Figure 3 illustrates Theorem 2 for $7x + 3y \leq 17$ and $m = 8$. Then $S = \lfloor 17/(8/2) \rfloor = 4$ and $[\![7x + 3y \leq 17]\!]_{\mathsf{LIA}} = [\![\phi_0 \vee \phi_1 \vee \phi_2]\!]_{\mathsf{BV}}$ where

$$\begin{aligned} \phi_0 &= 7x + 3y - \phantom{1}8 \leq 3 \wedge \mathrm{box}_{\mathsf{BV}}(\boldsymbol{c}; 11) \\ \phi_1 &= 7x + 3y - 12 \leq 3 \wedge \mathrm{box}_{\mathsf{BV}}(\boldsymbol{c}; 15) \\ \phi_2 &= 7x + 3y - 16 \leq 1 \wedge \mathrm{box}_{\mathsf{BV}}(\boldsymbol{c}; 17) \end{aligned}$$

The $\mathrm{box}_{\mathsf{BV}}(\boldsymbol{c}; 11), \mathrm{box}_{\mathsf{BV}}(\boldsymbol{c}, 15), \mathrm{box}_{\mathsf{BV}}(\boldsymbol{c}; 17)$ formulae are again depicted in grey. For example,

$$\mathrm{box}_{\mathsf{BV}}(\boldsymbol{c}; 11) = (x \leq 0 \wedge y \leq 3) \vee (x \leq 1 \wedge y \leq 2) \vee (x \leq 1 \wedge y \leq 1)$$

because $d = 2$, $L = 3$ and $I_2((d-1)(L+1)) = \{\langle 1, 3\rangle, \langle 2, 2\rangle, \langle 3, 1\rangle\}$. From Figure 3 observe $[\![7x + 3y \leq 17]\!]_{\mathsf{LIA}} = [\![\phi_0]\!]_{\mathsf{BV}} \cup [\![\phi_1]\!]_{\mathsf{BV}} \cup [\![\phi_2]\!]_{\mathsf{BV}}$.

*Example 7.* Consider again example 5 where $S = 1$. Then $\phi_0 = \mathit{false}$ because $\mathrm{box}_{\mathsf{BV}}(\boldsymbol{c}; (S-1)(m/2) - 1) = \mathrm{box}_{\mathsf{BV}}(\boldsymbol{c}; -1) = \mathit{false}$. This is because $L = 0$ and $I_d((d-1)(L+1)) = I_2(1) = \emptyset$. Theorem 2 then gives $[\![x + 2y \leq 5]\!]_{\mathsf{LIA}} = [\![\phi_1 \vee \phi_2]\!]_{\mathsf{BV}}$ which squares with Corollary 2.

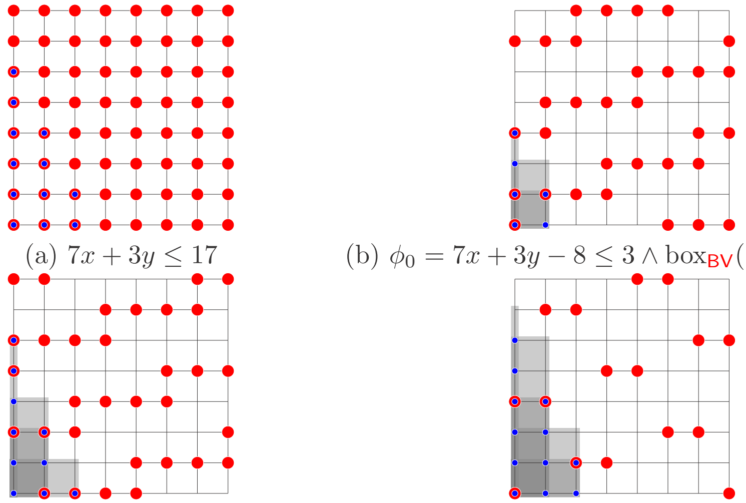

(a) $7x + 3y \leq 17$ (b) $\phi_0 = 7x + 3y - 8 \leq 3 \wedge \mathrm{box}_{\mathsf{BV}}(\boldsymbol{c}; 11)$

(c) $\phi_1 = 7x + 3y - 12 \leq 3 \wedge \mathrm{box}_{\mathsf{BV}}(\boldsymbol{c}; 15)$ (d) $\phi_2 = 7x + 3y - 16 \leq 1 \wedge \mathrm{box}_{\mathsf{BV}}(\boldsymbol{c}; 17)$

**Fig. 3.** Gapping and boxing for $7x + 3y \leq 17$ where $\boldsymbol{c} = \langle 7, 3 \rangle$, $m = 8$ and $S = 4$

### 3.3 Boxing, Gapping and Flipping

To handle inequalities which have indeterminates with negative coefficients, boxing and gapping are augmented with a third technique, which we have informally named flipping. Flipping transforms an inequality into a syntactic form which is amenable to boxing and gapping by reflecting the solutions of the inequality. To detail the transformation, we assume without loss of generality, that an inequality takes the syntactic form $\boldsymbol{c}^+ \cdot \boldsymbol{x}^+ + \boldsymbol{c}^- \cdot \boldsymbol{x}^- \leq b$ where $\boldsymbol{c}^+ > \boldsymbol{0}$ and $\boldsymbol{c}^- < \boldsymbol{0}$. Hence $\boldsymbol{x} = \boldsymbol{x}^+ \circ \boldsymbol{x}^-$ where $\circ$ denotes vector concatenation. The act of flipping reflects the solutions of the inequality simultaneously around the axes $x_1^- = 0, \ldots, x_e^- = 0$ where $\boldsymbol{x}^- = \langle x_1^-, \ldots, x_e^- \rangle$ and $e$ is the dimension of $x^-$. The development starts with the flipping transformation itself:

**Definition 3.** Given $e \in \{1, \ldots, d\}$, then the (semantic) flipping function $F_e : \mathbb{M}^d \to \mathbb{M}^d$ is defined:

$$F_e(\langle x_1^+, \ldots, x_{d-e}^+, x_1^-, \ldots, x_e^- \rangle) = \langle x_1^+, \ldots, x_{d-e}^+, m - 1 - x_1^-, \ldots, m - 1 - x_e^- \rangle.$$

Given an inequality with negative coefficients, we derive a new inequality whose solutions coincide with the flipped solutions of the given inequality. This transformation is then lifted to formulae as follows:

**Definition 4.** Given a partition of $\boldsymbol{x}$ into the sub-vectors $\boldsymbol{x}^+ = \langle x_1^+, \ldots, x_{d-e}^+ \rangle$ and $\boldsymbol{x}^- = \langle x_1^-, \ldots, x_e^- \rangle$, then the (syntactic) flipping function $F_{\boldsymbol{x}^-}$ is defined:

$$\begin{aligned} F_{\boldsymbol{x}^-}(\boldsymbol{c}^+ \cdot \boldsymbol{x}^+ + \boldsymbol{c}^- \cdot \boldsymbol{x}^- \leq b) &= \boldsymbol{c}^+ \cdot \boldsymbol{x}^+ - \boldsymbol{c}^- \cdot \boldsymbol{x}^- + (m-1)(\boldsymbol{c}^- \cdot \boldsymbol{1}) \leq b \\ F_{\boldsymbol{x}^-}(f_1 \vee f_2) &= F_{\boldsymbol{x}^-}(f_1) \vee F_{\boldsymbol{x}^-}(f_2) \\ F_{\boldsymbol{x}^-}(f_1 \wedge f_2) &= F_{\boldsymbol{x}^-}(f_1) \wedge F_{\boldsymbol{x}^-}(f_2) \\ F_{\boldsymbol{x}^-}(\neg f) &= \neg F_{\boldsymbol{x}^-}(f) \end{aligned}$$

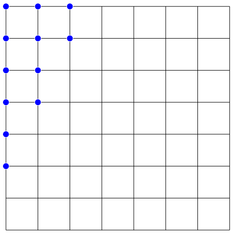

(a) $[\![\phi]\!]_{\mathsf{LIA}}$

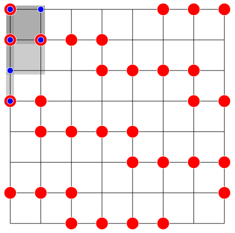

(b) $F_{\langle y \rangle}(\phi_0) = 7x - 3y + 13 \leq 3 \wedge F_{\langle y \rangle}(\mathrm{box}_{\mathsf{BV}}(\boldsymbol{c}; 11))$

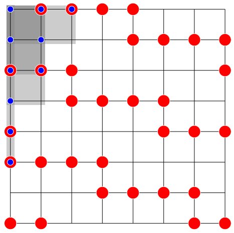

(c) $F_{\langle y \rangle}(\phi_1) = 7x - 3y - 9 \leq 3 \wedge F_{\langle y \rangle}(\mathrm{box}_{\mathsf{BV}}(\boldsymbol{c}; 15))$

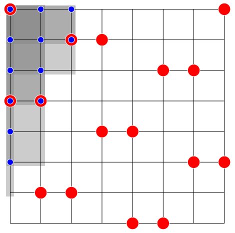

(d) $F_{\langle y \rangle}(\phi_2) = 7x - 3y + 5 \leq 1 \wedge F_{\langle y \rangle}(\mathrm{box}_{\mathsf{BV}}(\boldsymbol{c}; 17))$

**Fig. 4.** Flipping $\phi = 7x - 3y \leq -4$ where $m = 8$, $\boldsymbol{x} = \langle x, y \rangle$, $\boldsymbol{x}^+ = \langle x \rangle$ and $\boldsymbol{x}^- = \langle y \rangle$

The overall strategy involves applying boxing and gapping to an inequality derived by the flipping function $F_{\boldsymbol{x}^-}$. The validity of this strategy is based on the following proposition:

**Proposition 2.** If $|\boldsymbol{x}^-| = e$ then

- $[\![F_{\boldsymbol{x}^-}(f)]\!]_{\mathsf{LIA}} = F_e([\![f]\!]_{\mathsf{LIA}})$
- $[\![F_{\boldsymbol{x}^-}(f)]\!]_{\mathsf{BV}} = F_e([\![f]\!]_{\mathsf{BV}})$

A complete strategy for handling inequalities with negative coefficients is justified by the following corollary. The strategy entails flipping an LIA inequality, deriving a BV formula by boxing and gapping, and then flipping the BV formula.

**Corollary 3.** Suppose $\boldsymbol{c}^+ > \boldsymbol{0}$, $\boldsymbol{c}^- < \boldsymbol{0}$ and

$$[\![\boldsymbol{c}^+ \cdot \boldsymbol{x}^+ - \boldsymbol{c}^- \cdot \boldsymbol{x}^- \leq b + (1 - m)(\boldsymbol{c}^- \cdot \boldsymbol{1})]\!]_{\mathsf{LIA}} = [\![\phi_0 \vee \phi_1 \vee \phi_2]\!]_{\mathsf{BV}}$$

Then $[\![\boldsymbol{c}^+ \cdot \boldsymbol{x}^+ + \boldsymbol{c}^- \cdot \boldsymbol{x}^- \leq b]\!]_{\mathsf{LIA}} = [\![F_{\boldsymbol{x}^-}(\phi_0) \vee F_{\boldsymbol{x}^-}(\phi_1) \vee F_{\boldsymbol{x}^-}(\phi_2)]\!]_{\mathsf{BV}}$

*Example 8.* Consider $\phi = 7x - 3y \leq -4$ which is illustrated in Fig. 4(a). Then $\boldsymbol{x}^+ = \langle x \rangle$, $\boldsymbol{x}^- = \langle y \rangle$ and $F_{\boldsymbol{x}^-}(\phi) = F_{\langle y \rangle}(\phi) = 7x + 3y - 21 \leq -4$. Fig. 3(a)

shows $[\![7x+3y-21 \leq -4]\!]_{\mathsf{LIA}} = [\![7x+3y \leq 17]\!]_{\mathsf{LIA}}$ and so building on example 6 $[\![7x+3y \leq 17]\!]_{\mathsf{LIA}} = [\![\phi_0 \vee \phi_1 \vee \phi_2]\!]_{\mathsf{BV}}$. By corollary 3 it follows $[\![\phi]\!]_{\mathsf{LIA}} = [\![F_{\langle y \rangle}(\phi_0) \vee F_{\langle y \rangle}(\phi_1) \vee F_{\langle y \rangle}(\phi_2)]\!]_{\mathsf{BV}}$ where $F_{\langle y \rangle}(\phi_0)$, $F_{\langle y \rangle}(\phi_1)$ and $F_{\langle y \rangle}(\phi_2)$ are given in Fig. 4(b), (c) and (d) respectively. Finally, to illustrate the handling of boxing, recall $\text{box}_{\mathsf{BV}}(\boldsymbol{c}; 11)$ from example 6 and

$$\begin{aligned} \text{box}_{\mathsf{BV}}(\boldsymbol{c}; 11) &= (x \leq 0 \wedge y \leq 3) \\ &\vee (x \leq 1 \wedge y \leq 2) \\ &\vee (x \leq 1 \wedge y \leq 1) \end{aligned} \qquad \begin{aligned} F_{\langle y \rangle}(\text{box}_{\mathsf{BV}}(\boldsymbol{c}; 11)) &= (x \leq 0 \wedge (-y+7 \leq 3)) \\ &\vee (x \leq 1 \wedge (-y+7 \leq 2)) \\ &\vee (x \leq 1 \wedge (-y+7 \leq 1)) \end{aligned}$$

Finally observe

$$\begin{aligned} [\![x \leq 0 \wedge (-y+7 \leq 3)]\!]_{\mathsf{LIA}} &= \{(0, y) \in \mathbb{M}^2 \mid 4 \leq y \leq 7\} \\ [\![x \leq 1 \wedge (-y+7 \leq 2)]\!]_{\mathsf{LIA}} &= \{(x, y) \in \mathbb{M}^2 \mid 0 \leq x \leq 1 \wedge 5 \leq y \leq 7\} \end{aligned}$$

and that the disjunct $(x \leq 1 \wedge (-y+7 \leq 1))$ is actually redundant.

### 3.4 Boxing, Gapping, Flipping and Demoding

Griggio [16] gives a procedure for encoding machine arithmetic in LIA, illustrating that the resulting LIA interpolants can include inequalities such as $-x_2+x_3-256\lfloor -x_2/256 \rfloor \leq 255$ [16, Example 5]. Relaxing inequalities to include ceiling (or floor) functions can reduce the size of interpolants whilst simplifying their derivation [17]. These more general forms of interpolant include inequalities of the form $\boldsymbol{c} \cdot \boldsymbol{x} + n' \lfloor \boldsymbol{c}' \cdot \boldsymbol{x}/n \rfloor \leq b$ [9] or $\boldsymbol{c} \cdot \boldsymbol{x} + n' \lceil \boldsymbol{c}' \cdot \boldsymbol{x}/n \rceil \leq b$ [17], though for our purposes it is sufficient to consider $\boldsymbol{c} \cdot \boldsymbol{x} + n' 2^n \lfloor \boldsymbol{c}' \cdot \boldsymbol{x}/2^n \rfloor \leq b$ or $\boldsymbol{c} \cdot \boldsymbol{x} + n' 2^n \lceil \boldsymbol{c}' \cdot \boldsymbol{x}/2^n \rceil \leq b$, where the divisors are powers of 2, stemming from the way they model wrap-around in machine arithmetic. To extend boxing to these generalised interpolants we extend the LIA and BV semantics two new types of atomic constraint (though the definitions are almost vacuous):

**Definition 5.** *If* $\ell \equiv \boldsymbol{c} \cdot \boldsymbol{x} + n' \lfloor \boldsymbol{c}' \cdot \boldsymbol{x}/2^n \rfloor \leq b$ *then*

$$\begin{aligned} [\![\ell]\!]_{\mathsf{LIA}} &= \left\{ \boldsymbol{x} \in \mathbb{M}^d \middle| \boldsymbol{c} \cdot \boldsymbol{x} + n' \lfloor \boldsymbol{c}' \cdot \boldsymbol{x}/2^n \rfloor \leq b \right\} \\ [\![\ell]\!]_{\mathsf{BV}} &= \left\{ \boldsymbol{x} \in \mathbb{M}^d \middle| (\boldsymbol{c} \cdot \boldsymbol{x} + n' \lfloor \boldsymbol{c}' \cdot \boldsymbol{x}/2^n \rfloor) \bmod m \leq b \bmod m \right\} \end{aligned}$$

The following proposition shows generalised LIA interpolants are not an obstacle to boxing. These inequalities are handled through a transformation scheme which exploits the property that if $n \leq w$ then $(\boldsymbol{c} \cdot \boldsymbol{x} \bmod 2^n) \bmod m = \boldsymbol{c} \cdot \boldsymbol{x} \bmod 2^n$. We informally call this transformation tactic demoding, because like gapping and flipping, it is designed to increase the general applicability of boxing.

**Proposition 3.** Suppose $0 \leq n \leq w$ and $[\![(\boldsymbol{c} + n'\boldsymbol{c}') \cdot \boldsymbol{x} - n'y \leq b]\!]_{\mathsf{LIA}} = [\![\phi]\!]_{\mathsf{BV}}$. If $y$ does not occur in $\boldsymbol{x}$ then

$$[\![\boldsymbol{c} \cdot \boldsymbol{x} + n' 2^n \lfloor \boldsymbol{c}' \cdot \boldsymbol{x}/2^n \rfloor \leq b]\!]_{\mathsf{LIA}} = [\![\phi[y \mapsto \boldsymbol{c}' \cdot \boldsymbol{x} \bmod 2^n]]\!]_{\mathsf{BV}}$$

Inequalities such as $\boldsymbol{c} \cdot \boldsymbol{x} + n' 2^n \lceil \boldsymbol{c}' \cdot \boldsymbol{x}/2^n \rceil \leq b$ can be handled similarly. For completeness, we note that expansion can be applied for non-powers of 2:

**Proposition 4.** Suppose $n > 0$. Then

$$[\![\boldsymbol{c} \cdot \boldsymbol{x} + n' \lfloor \boldsymbol{c}' \cdot \boldsymbol{x}/n \rfloor \leq b]\!]_{\mathsf{LIA}} = [\![\bigvee_{i=\ell}^{u} (\boldsymbol{c} \cdot \boldsymbol{x} \leq b - n'i \wedge ni \leq \boldsymbol{c}' \cdot \boldsymbol{x} \leq ni - 1)]\!]_{\mathsf{LIA}}$$

where $\ell = \min\{\lfloor \boldsymbol{c}' \cdot \boldsymbol{x}/n \rfloor \mid \boldsymbol{x} \in \mathbb{M}^d\}$ and $u = \max\{\lfloor \boldsymbol{c}' \cdot \boldsymbol{x}/n \rfloor \mid \boldsymbol{x} \in \mathbb{M}^d\}$.

## 4 Experiments

To evaluate the performance of boxing we implemented a model checker based on the lazy abstraction (IMPACT) [25] algorithm. The model checker is implemented in Python 3.7.2 and uses MathSAT5 [9] for satisfiability checking and interpolation over LIA. The model checker parses a subset of the C language, but is rich enough to handle 312 benchmarks drawn from [2, 12]. The model checker was instantiated in one of three ways to use: (1) LIA interpolation [17]; (2) BV interpolation by covering the solutions of an LIA interpolate with columns (recall $f_2$ of section 2); and (3) BV interpolation by covering the solutions of an LIA interpolate using boxing, gapping and flipping. Experiments were performed using an Amazon Web Service EC2 c3.xlarge cloud architecture of 14 EC2 Computing Units [31] each equipped with 4 cores and 7.5 GB of RAM. The timeout for each run of IMPACT was set to 600 seconds.

All arithmetic is idealised in configuration (1) taking no account of integer overflow and underflow. This is not, in general, safe. In configurations (2) and (3) the model checker interprets machine arithmetic and bit operations using the LIA encoding of BV operations outlined in [16, Fig 1]. This is safe but complicates the LIA formulae, often substantially. One would expect this to enlarge the interpolants, even before boxing and gapping are deployed. We would also expect (1) to be substantially faster than (2) and (3). Due to differences in the semantics of arithmetic, we might also see differences in the number of programs proved to be safe or found to be unsafe. The experiments quantify these predictions. To discuss the experiments, (2) will be referred to as the naive encoding, even though it improves on complete enumeration (recall $f_1$ of section 2).

### 4.1 Overall Result

Table 4.1 summarises the outcomes of running IMPACT on all 312 programs, using the three different instances of interpolation, categorised as to whether the run proved safety (safe rows) or found a counterexample (unsafe rows). The Solved column of the left-hand table gives the total of the programs there were either shown to be safe or unsafe within 600 seconds. Time is the mean execution of a run (for all those programs which did not timeout). Size is mean

**Table 1.** Comparison of the theories: performance and correctness

| Theory | Safety | Solved | Time (seconds) | Size (inequalities) |
|---|---|---|---|---|
| LIA | safe | 165 | 15.1 | 440 |
| | unsafe | 41 | 9.0 | 392 |
| | (total) | 206 | 13.9 | 431 |
| BV (naive) | safe | 87 | 30.1 | 32583 |
| | unsafe | 57 | 24.2 | 49138 |
| | (total) | 144 | 27.8 | 39136 |
| BV (boxing) | safe | 99 | 20.0 | 6938 |
| | unsafe | 66 | 20.1 | 15246 |
| | (total) | 165 | 20.0 | 10261 |

| | | LIA | |
|---|---|---|---|
| | | safe | unsafe |
| BV | safe | 90 | 1 |
| | unsafe | 17 | 34 |

total number of atomic constraints in all interpolants encountered over a run (for those programs which did not timeout). We observe that more programs can be analysed to completion with LIA than with BV, as one would expect, but that BV (boxing) improves on BV (naive), the speedup being significant when proving safety.

The right-hand table compares a terminating run of LIA to a terminating run of BV (boxing). For 17 of these 142 runs, LIA (incorrectly) verified the program to be safe whereas BV found a counter-example. Unexpectantly for trex03_true-unreach-call.i.annot.c from [12], LIA found a counter-example but BV verified safety. This program contains three integers, `x1`, `x2` and `x3`, which can become negative in the idealised arithmetic employed in LIA, triggering an assertion. But `x1`, `x2` and `x3` are actually unsigned.

### 4.2 Runtime for Naive encoding and Boxing

The scatter plot of Figure 5 compares the runtime of the naive encoding against that of boxing and its allied techniques of gapping and flipping. The scatter plot excludes timeouts and depicts 151 pairs of runs. Almost all points are under the dotted line, indicating the boxing significantly improves performance. The line graph plots the ratio of the execution times, from which we observe that boxing does not accelerate the verification for almost half of the runs, but does speed it up between 2- and 256-fold for the other half.

### 4.3 Interpolant Size for Naive encoding and Boxing

The line graph on Figure 6 compares the relative size of interpolants for boxing versus the naive encoding. Size is the sum of the sizes of all the interpolants generated during a run, where the size of an interpolant is itself defined as the number of atomic constraints that occur within it. We observe that for most problems the size ratio is around one, but a second peak occurs at 1/32, giving an overall size reduction. The scatter plot explores how interpolant size correlates with runtime, showing how the relative size of interpolants varies with relative runtimes. We observe that reducing the size of interpolants improves runtime, and that two peaks of the line graph manifesting themselves as two clusters of points in the scatter plot.

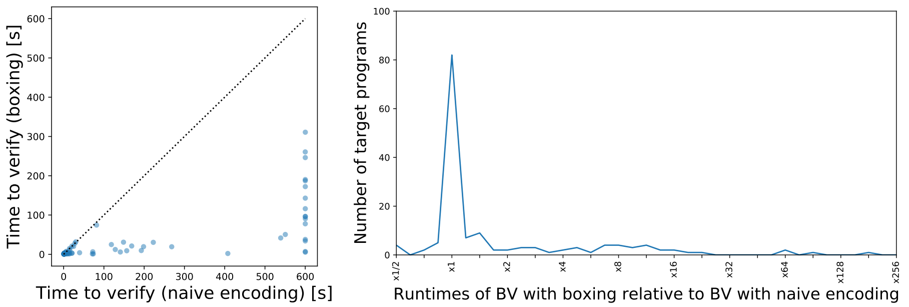

**Fig. 5.** Runtime of boxing versus naive: scatter plot and ratio plot

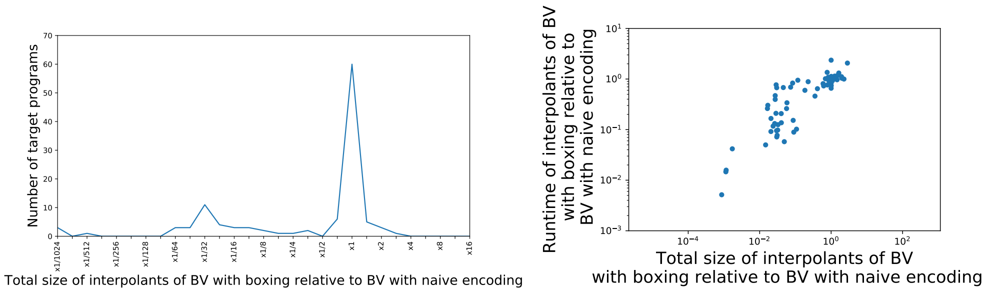

**Fig. 6.** Size of interpolants in boxing versus naive and its impact on performance

## 5 Related work

The problem of reasoning about machine arithmetic and wrapping arises not only in model checking, but abstract interpretation too, where solvers are augmented with support for relaxing abstractions by join rather than interpolation.

Despite the long-standing work [3, 7, 27] in deciding BV theories, there has been scant work on BV interpolation. Although not focussing on BV interpolation, an early work on deriving work-level interpolants [23] uses bit-vectors to interpolate equality logic. This logic supports equations of the form $x = y$ and $x = c$ where $x$ and $y$ are variables and $c$ is drawn from a finite set of symbols $C$. Bit-vectors with width $\lceil \log_2(|C|) \rceil$ are used to bit-blast equations [29] so that formulae are encoded entirely propositionally. Then a propositional resolution proof of the inconsistence of two formulae is lifted to the work-level.

Seminal work by Griggio [16] advocated encoding BV formulae in theories of increasing complexity. The pair of BV formulae are encoded in a theory whose interpolation engine is used to find an interpolant in that theory. The interpolant is then reinterpreted as a BV formula and tested to see if it is still an interpolant the pair of BV formulae. The approach resorts to bit-blasting if no simpler theory can find an interpolant, at the cost of losing world-level information. By way of contrast, Backeman et al. [2] propose a calculus over a core language, which supports interpolation and is rich enough to describe BV formulae, even making use of Groebner bases to express polynomial equality relationships. Since interpolation is performed within their core language, they do not aim to derive a BV interpolant, and therefore their work is orthogonal to ours. Yet if Backeman's procedure returns an interpolant in their core language and it could be interpreted as an LIA formula, which would seem likely for many cases, then our work could convert the LIA formula back to BV.

Further afield, polynomial algorithms for interpolation have developed for systems of linear congruence equations [19, section 4], conjunctions of linear Diophantine equations and disequations [19, section 6], and systems of mixed integer linear equations [19, section 7]. This comprehensive study stops short of using LIA to interpolate BV formula, mentioning the problem as future work.

Abstract domains have been proposed for tracking linear modulo relationships where the module is a power of 2 [13, 22, 28]. These domains, which are essentially specialist solvers, express more than linear equalities [21], while enabling the domain operations to be realised using machine arithmetic. Surprisingly, systems of linear inequalities can be reinterpreted to model machine arithmetic by just changing the concretisation function [32] and the handling of guards [32].

## 6 Concluding Discussion

To repurpose efficient LIA interpolation engines to BV, we have shown how to systematically construct a BV formula so its solutions are exactly those of an LIA interpolant. Since an LIA interpolant summarises the reason for a conflict between two LIA formula, we seek to retain its compact structure by introducing no more than simple boxes around the LIA solutions which block extraneous BV solutions. When this encoding tactic, called boxing, is not applicable, gapping is used to decompose an LIA inequality into two or more inequalities which are amenable to boxing. We show how the size of the resulting BV interpolants are smaller than BV interpolants constructed by merely partitioning the LIA solutions into columns, and demonstrate how boxing and gapping improves the runtime of an interpolation-based model-checker. We instantiate a model-checker with LIA and BV to compare their performance, and conclude that with this encoding BV interpolation is feasible. Because of wrap-around, BV is substantially more complicated than LIA for interpolation, yet BV is no more than twice as slow as LIA for over half the benchmarks. Furthermore, the resulting BV interpolants can be validated, independent of LIA, just using a BV solver.

*Acknowledgments* We thank anonymous reviewers for their comments which helped us improve the paper. We thank Alberto Griggio for his help with MathSAT5 and looking into the intellectual property restrictions on sharing his extension to Kratos [8], a model checker based on lazy predicate abstraction. We also thank Chris Coppins with his help with IMPACT and Peter Backeman for tirelessly answering our e-mails. This work was funded, in part, by EPSRC EP/N020243/1 and by JST ERATO HASUO Metamathematics for Systems Design Project JPMJER1603.
